# Differential Expression of Putative *Ornithodoros turicata* Defensins Mediated by Tick Feeding

**DOI:** 10.3389/fcimb.2020.00152

**Published:** 2020-05-05

**Authors:** Brittany A. Armstrong, Alexander R. Kneubehl, Robert D. Mitchell, Aparna Krishnavajhala, Pete D. Teel, Adalberto A. Pérez de León, Job E. Lopez

**Affiliations:** ^1^Department of Molecular Virology and Microbiology, Baylor College of Medicine, Houston, TX, United States; ^2^Department of Pediatrics, National School of Tropical Medicine, Baylor College of Medicine, Houston, TX, United States; ^3^Knipling-Bushland U.S. Livestock Insects Research Laboratory, Veterinary Pest Genomics Center, Department of Agriculture—Agricultural Research Service, Kerrville, TX, United States; ^4^Department of Entomology, Texas A&M AgriLife Research, College Station, TX, United States

**Keywords:** *Ornithdoros turicata*, antimicrobial peptide (AMP), gene expression, defensins, argasid (soft) ticks, immune response

## Abstract

Additional research on soft ticks in the family Argasidae is needed to bridge the knowledge gap relative to hard ticks of the family Ixodidae; especially, the molecular mechanisms of *Ornithodoros* biology. *Ornithodoros* species are vectors of human and animal pathogens that include tick-borne relapsing fever spirochetes and African swine fever virus. Soft tick vector-pathogen interactions involving components of the tick immune response are not understood. Ticks utilize a basic innate immune system consisting of recognition factors and cellular and humoral responses to produce antimicrobial peptides, like defensins. In the present study, we identified and characterized the first putative defensins of *Ornithodoros turicata*, an argasid tick found primarily in the southwestern United States and regions of Latin America. Four genes (*otdA, otdB, otdC*, and *otdD*) were identified through sequencing and their predicted amino acid sequences contained motifs characteristic of arthropod defensins. A phylogenetic analysis grouped these four genes with arthropod defensins, and computational structural analyses further supported the identification. Since pathogens transmitted by *O. turicata* colonize both the midgut and salivary glands, expression patterns of the putative defensins were determined in these tissues 1 week post engorgement and after molting. Defensin genes up-regulated in the tick midgut 1 week post blood feeding were *otdA* and *otdC*, while *otdD* was up-regulated in the midgut of post-molt ticks. Moreover, *otdB* and *otdD* were also up-regulated in the salivary glands of flat post-molt ticks, while *otdC* was up-regulated within 1 week post blood-feeding. This work is foundational toward additional studies to determine mechanisms of vector competence and pathogen transmission from *O. turicata*.

## Introduction

*Ornithodoros* (argasid) species are vectors of veterinary and medically significant pathogens. The primary species in the United States that transmit pathogens include *Ornithodoros turicata, Ornithodoros hermsi, Ornithodoros parkeri, Ornithodoros talaje*, and *Ornithodoros coriaceus* (Davis, 1939; Cooley and Kohls, 1944; Hess et al., 1987; Donaldson et al., 2016; Lopez et al., 2016; Sage et al., 2017). These species have been implicated in the transmission of tick-borne relapsing fever spirochetes (Lane et al., 1985; Dworkin et al., 2002; Nieto et al., 2012; Lopez et al., 2016; Christensen et al., 2017; Bissett et al., 2018). Moreover, *O. turicata* and *O. coriaceus* were experimentally shown to be competent vectors of African swine fever virus (ASFV) (Hess et al., 1987), an emerging pathogen in Europe and Asia. *O. parkeri* was able to be infected with ASFV, but unable to transmit the pathogen via tick bite (Hess et al., 1987). *Ornithodoros* ticks play a significant role in pathogen maintenance, yet very little is known regarding vector competence.

The life cycles of *Ornithodoros* ticks and their pathogens has been predominantly characterized utilizing *O. hermsi* and *O. turicata* models, and these studies identified significant challenges when attempting to elucidate mechanisms of vector competence. *Ornithodoros* species are rapid feeders, completing the bloodmeal within 5–60 min (Balashov, 1972). As ticks blood feed, pathogens initially colonize the midgut (Schwan, 1996; Schwan and Hinnebusch, 1998; Krishnavajhala et al., 2017). Given that transmission can occur within seconds of tick attachment, pathogens must also colonize the salivary glands to ensure entrance into a vertebrate host (Schwan and Hinnebusch, 1998; Boyle et al., 2014; Krishnavajhala et al., 2017). *Ornithodoros* species are also unique from other tick genera because they live for 10–20 years and can endure over 5 years of starvation and still remain competent vectors (Davis, 1943; Assous and Wilamowski, 2009). Recently, physiological differences were detected between the midgut and salivary glands of *O. turicata*, which revealed selective pressures that pathogens encounter in the argasid tick including reactive nitrogen and oxygen species (ROS and RNS) (Bourret et al., 2019). However, it remains vague what other immunological pressures exist in these two disparate environments.

Three identified branches of tick immunity are currently recognized: immune regulation components (recognition factors and signaling pathways), cellular, and humoral (Kopáček et al., 2010; Hynes, 2014). In argasid ticks, identified recognition factors include lectins, which are important for self and non-self-recognition (Grubhoffer et al., 2004; Kopáček et al., 2010). Cellular responses have primarily been found in the hemocoel and include hemocyte responses and phagocytosis of pathogens (Sonenshine et al., 2002; Nakajima et al., 2003b; Oliva Chavez et al., 2017; Sonenshine and Macaluso, 2017). The most characterized portion of soft tick immunity, though still significantly understudied, is humoral immunity. Within this branch of immunity are proteases, protease inhibitors, and antimicrobial peptides (AMPs) (Sonenshine and Hynes, 2008; Hajdusek et al., 2013; Oliva Chavez et al., 2017).

AMPs are a broad category of immune molecules that function to protect the vector from pathogens (Sonenshine et al., 2002; Boulanger et al., 2006; Hajdusek et al., 2013; Tonk et al., 2014). AMPs include lysozymes, hebraein, microplusin, ixodidin, ixosin, *Ixodes scapularis* AMP (isAMP), hemoglobin fragments, and defensins (Grunclová et al., 2003; Lai et al., 2004; Sonenshine et al., 2005; Fogaça et al., 2006; Liu et al., 2008; Silva et al., 2009; Chrudimska et al., 2010; Hajdusek et al., 2013). A key component of tick and other arthropod immunity are defensins. These are cationic molecules that disrupt the cell membrane of pathogens by binding to the negatively charged membrane and forming a pore leading to cell depolarization and ultimately cell death (Nakajima et al., 2003a; Bulet and Stöcklin, 2005). Tick defensins consist of a signal peptide, pro-segment containing a furin cleavage site (RVRR) (Chrudimska et al., 2014), and the mature peptide (Hosaka et al., 1991; Nakajima et al., 2001). The mature peptide is characterized with six cysteine residues that form three disulfide bonds resulting in a cysteine-stabilized αβ (CSαβ) motif (Bulet and Stöcklin, 2005). Proper cysteine pairing through disulfide bonds is crucial for antimicrobial activity (Isogai et al., 2011).

In this study, we focused on identifying immunological pressures produced in the tick midgut and salivary glands. Since genomic and transcriptomic resources are limited for *O. turicata*, we utilized a salivary gland transcriptome to identify putative defensins (Bourret et al., 2019). These candidates had the characteristic six cysteine residues observed in known defensin molecules of insects and arthropods. Transcripts were further evaluated by rapid amplification of cDNA ends (RACE) to obtain full-length sequences. We performed computational analyses at the protein level to generate predictive structures, which further supported the characterization of theses transcripts as defensins. A phylogenetic analysis was also performed with defensins from numerous arthropod species. Lastly, we investigated expression patterns of these putative defensins in the midgut and salivary gland tissues 1 week after *O. turicata* fed and after the molt. These time points were chosen because of their importance in early and post-molt pathogen colonization. Our initial findings suggest that in *Ornithodoros* species defensins may have a role directly after blood feeding, while others are utilized in post-molt ticks. Our findings provide a foundation to further investigate the molecular mechanisms of vector competence in a rapid feeding, long-lived tick.

## Materials and Methods

### Identification of Defensins and RACE Sequencing

*O. turicata* defensins were identified from our previously reported salivary gland transcriptome (Bourret et al., 2019). The transcriptome was analyzed to select transcripts that were annotated as defensins. Putative defensins were evaluated in National Center for Biotechnology Information (NCBI) using Basic Local Alignment Search Tool (BLAST) to confirm the transcriptome results by assessing amino acid sequence homology with other arthropod defensins (Altschul et al., 1990).

Rapid amplification of cDNA ends (RACE) was performed with mRNA extracted from a pool of 9 ticks 9 days post blood feeding, using the Nucleotrap mRNA MiniKit (Takara Bio Inc, Kusatsu, Japan) and following the manufacturer's instructions. Purified mRNA was used with the SMARTer RACE 5'/3' kit (Takara Bio Inc, Kusatsu, Japan) and for both the 5' and 3' ends of the defensin transcripts. Gene-specific oligos for 5' and 3' RACE (Table 1) were designed according to manufacturer's instructions based on sequences identified as defensins in the RNA-seq dataset and were manufactured by Sigma-Aldrich (St. Louis, MO). The 5' and 3' RACE reactions were performed following the manufacturer's instructions using the Touchdown PCR protocol. The only modification was the extension times throughout the protocol were shortened to 1 min. RACE PCR reactions were analyzed by agarose gel electrophoresis (0.8% agarose tris-acetate EDTA buffer) and positive PCR reactions were purified using the QIAquick PCR purification kit (Qiagen, Hilden, Germany). Following manufacturers' instructions, purified PCR products were cloned using the Zero Blunt TOPO PCR Cloning Kit for Sequencing (ThermoFisher, Waltham, MA) and used to transform NEB10-beta chemically competent cells (New England BioLabs, Ipswich, MA). Transformants were plated on LB-Miller agar (BD Biosciences, San Jose, CA) containing 50 ug/mL of kanamycin (Sigma-Aldrich, St. Louis, MO) overnight at 37°C. Colonies were screened by PCR with universal M13F/R primers (Sigma-Aldrich, St. Louis, MO) at a final concentration of 200 nM and using OneTaq 2x PCR mastermix with standard buffer (New England BioLabs, Ipswich, MA) using the following colony PCR protocol: 94°C for 5 min: 1 cycle; 94°C 30 s, 50°C 30 s, 68°C 40 s: 25 cycles; 68°C 5 min: 1 cycle; 10°C hold. Colony PCR was analyzed by agarose gel electrophoresis and the colonies with the largest products were selected for sequencing. Selected colonies were cultured overnight in 5 mL of LB-Miller with 50 ug/mL of kanamycin and plasmid isolated the following day with the QiaPrep Spin Miniprep Kit (Qiagen). Purified RACE product plasmids were sent to GeneWiz (Plainfield, NJ) for Sanger sequencing using M13F/R sequencing primers. Sequencing results were analyzed using FinchTV (v1.4.0, Digital World Biology) and the predicted amino acid sequences were BLASTed (Blastp) against representative non-redundant protein sequences on NCBI to confirm their identity (Altschul et al., 1990). Complete coding sequences were submitted to Genbank under the names *otdA* (MN725028), *otdB* (MN725029), *otdC* (MN725030), and *otdD* (MN725031).

**Table 1:** Oligonucleotides and probes used for qPCR and RACE.

**Gene**	**Primer sequences (5′-3′)**
**qPCR primer sets**	
OtdA F	AGGACGGTACGGGGAAT
OtdA R	CGCACTTCTGGTCCAGC
OtdA Probe	YAK-ACCAGTACCAGTGCCACAGCCACTG-BBQ
OtdB F	AGGACGGTACGGGGAAT
OtdB R	CGCACTTCTGGTCCAGC
OtdB Probe	YAK-AGCCCGGTGCATCTTCCATATGC-BBQ
OtdC F	TGTTCTGAGTGCCGTTGTTAC
OtdC R	TGCTTCCGACACATAGCG
OtdC Probe	YAK-AGGCGTGCTCCGTGTTATGCAC-BBQ
OtdD F	TTTCGGTGTGCATTGTAGC
OtdD R	GGCAATGCTGATTGCACT
OtdD Probe	YAK-TGCAGATGGTGGCAGCGGCT-BBQ
BA F	TATCCACGAGACCACCTACAA
BA R	TCTGCATACGATCGGCAATAC
BA Probe	FAM-AAGGACCTGTACGCCAACACTGTC-IBFQ
**RACE primers**	
M13 F	GTAAAACGACGGCCAG
M13 R	CAGGAAACAGCTATGAC
OtdA 5′	AGTGGCTGTGGCACTGGTACTGG
OtdA 3′	AAGAGTCATCAGCCGTCCGAGTTCGT
OtdB 5′	CCTTTCGCACTTGAAGGTACAGGCAA
OtdB 3′	TGCTGCTTCTTACTGGGCTTCTCACTTC
OtdC 5′	AAGTCTCTACGCAGTGCTTCCGACACAT
OtdC 3′	CCCAGCAATGACTCCTCTCCGTTT
OtdD 5′	TCTGGTGTCGAGGCAATGCTGATT
OtdD 3′	CGGTGTGCATTCTAGCCCTCCTGC

*F, forward; R, reverse; YAK, Yakima dye; BBQ, blackberry quencher; FAM, fluorescein amide; IBFQ, Iowa Black FQ quencher*.

### Defensin Sequence Alignment and Protein Structure Prediction

The amino acid sequences of the identified *O. turicata* defensins were aligned with defensins published in Genbank from *Ornithodoros moubata* (BAB41028.1), *Carios puertoricensis* (ACJ04430.1), *I. scapularis* (XP_029834656.1), *Haemaphysalis longicornis* (ATN39848.1), *Dermacentor variabilis* (AAO24323.1), *Amblyomma americanum* (ABI74752.1), and *Argas monolakensis* (ABI52686.1). Mature peptide sequences were aligned using ClustalW in MEGAX 10.0.5 (Kumar et al., 2018). The signal peptide and propeptide cleavage sites were predicted using the ProP 1.0 Server (Duckert et al., 2004).

The structure of the defensins was predicted as described by Rodríguez-García et al. (2016). Briefly, protein model templates were identified based on sequence alignments generated with the Hhpred server (https://toolkit.tuebingen.mpg.de/tools/hhpred). The top three sequences were selected from tertiary structure prediction using MODELLER (https://toolkit.tuebingen.mpg.de/tools/modeller) (Webb and Sali, 2016; Zimmermann et al., 2018). The predicted structure was analyzed with ProSA-web (https://prosa.services.came.sbg.ac.at/prosa.php) to assess the Z-score (Wiederstein and Sippl, 2007). The final structure was generated using the Swiss-PDB Viewer 4.1.0 (Guex and Peitsch, 1997).

### Phylogenetic Analysis of Arthropod Defensins

All reported analyses were performed using NGPhylogeny.fr (Lemoine et al., 2018). Analyses with the same input data were also performed using MEGAX for comparison (data not shown) (Kumar et al., 2018). Alignments of the mature peptide sequence were performed using MUSLCE v3.8.31 under the “most accurate, maxiters = 16” run option setting and “UPGMB” under the clustering setting (Edgar, 2004). Noisy v1.5.12.1 was used for alignment curation with a cut-off threshold of 0.8 and the Hamming distance method via the Neighbor-Net ordering method (Dress et al., 2008). FastTree v2.1.10_1 was used to infer the phylogenetic tree with the WAG evolutionary model using Gamma distribution and 1,000 bootstraps (Price et al., 2009, 2010).

### Tick Colony Maintenance, Feedings, and Dissections

The present study used laboratory reared mid to late nymphal stage *O. turicata* ticks descendent of ticks originally collected in Travis County, Texas (Kim et al., 2017). The ticks were maintained in colony at 25°C and 80–85% humidity, as previously described (Lopez et al., 2013). Ticks were fed on an Institute of Cancer Research (ICR) mouse and dissected 1 week later (fed) or allowed to molt (post-molt). Each biological replicate consisted of 10 pooled midguts or salivary gland pairs from post-molt or fed ticks.

Midguts and salivary glands were extracted using an Axio Stemi dissection microscope (Zeiss, Munich, Germany). An individual tick was placed on a microscope slide in 10 to 20 μl of 1x Dulbecco's Phosphate Buffered Saline (DPBS) (Life Technologies, Grand Island, NY). The cuticle was removed, and the midgut extracted and placed in 100 μl of RNAlater (Qiagen, Hilden, Germany). The tick was then rinsed with 10 μl of 1x DPBS, the salivary glands removed and placed in 15 μl of 1x DPBS, rinsed, and placed in a tube containing 100 μl of RNAlater. Each sample consisted of 10 pooled midguts or salivary gland pairs. Samples were stored at −80°C until RNA was extracted.

### RNA Extraction, cDNA Synthesis, and RT-qPCR Analysis

Tissues were homogenized using a pestle (Argos Technologies, Elgin, IL) and were spun through a QIAshredder column per manufacturer's instructions (Qiagen, Hilden, Germany). RNA was extracted using the RNeasy mini kit (Qiagen, Hilden, Germany) following manufacturer's instructions. Samples were DNase treated (Qiagen, Hilden, Germany) and eluted in 30 μl of nuclease free water (Ambion, Inc, Austin, TX). RNA was quantified using a NanoDrop 2000 spectrophotometer (software v1.6.198, ThermoFisher Scientific, Waltham, MA).

RNA was converted to cDNA using the iScript cDNA synthesis kit (Bio-Rad, Hercules, CA) per the manufacturer's instructions. Gene expression was assessed using the cDNA to perform duplex qPCR. Primers against the defensins for qPCR (Table 1) were designed using the sequences from RACE sequencing and were synthesized by Integrated DNA Technologies (Coralville, IA). Defensins were run in duplex with *O. turicata* β*-actin* (Krishnavajhala et al., 2018), using the SsoAdvanced Universal Probes Supermix (Bio-Rad, Hercules, CA). Assays were performed with primers and probes at a concentration of 400 and 300 nM, respectively. The conditions for the assay were 50°C for 2 min (hold), 95°C for 3 min (polymerase activation), 95°C for 15 s (DNA denaturation), 60°C for 30 s (annealing and extension), repeating steps three (DNA denaturation) and four (annealing and extension) for 40 cycles on a CFX384 Touch Real-Time PCR Detection System (Bio-Rad, Hercules, CA). Each defensin was run with at least four biological replicates per tissue, with each replicate being run in technical triplicate.

### Statistical Analyses

The RT-qPCR data were statistically evaluated using Prism (Graphpad 8.2.1, San Diego, CA). Data were analyzed by normalizing the defensin genes to β*-actin* (ΔCt) and calculating the 2^−ΔCt^ of each reaction and performing a Student's *t*-test with Welch's correction to determine significance. In order to perform a statistical analysis, any gene that we could not detect expression in a given condition, the Ct value was set to 40 (the cutoff number of cycles). This was done for *otdC* in post-molt midguts and *otdD* in fed midguts. To determine the log_2_ fold change, the average fold change was calculating by dividing the 2^−ΔCt^ value by the average of the post-molt reactions 2^−ΔCt^ for that defensin and tissue. Subsequently, the log_2_ fold change for each reaction was determined and the mean and standard deviation calculated for fed and post-molt samples. Expression was significantly different if there was a log_2_ fold change of at least 1 (equivalent to a fold change of 2) and the *p*-value for the *t*-test was ≤ 0.05.

## Results

### Molecular Analysis of *O. turicata* Defensins

We evaluated the *O. turicata* salivary gland transcriptome (Bourret et al., 2019) with the goal of identifying defensin transcripts. Through this analysis we identified five candidates. RACE and Sanger sequencing validated the transcriptome results and confirm the full coding sequence of each defensin. We analyzed the full coding sequences by BLASTp and one candidate was omitted because the full-length cDNA failed to align to known arthropod defensins. The remaining four defensin genes were designated putative *O. turicata defensin A* (*otdA*), *B* (*otdB*), *C* (*otdC*), and *D* (*otdD*), and their sequences are in Table 2.

**Table 2:** Predicted cleavage sites of putative *O. turicata* defensins.

**Defensin**	**Amino acid sequence**
OtdA	MKTVFVIALVFALAVASMA^*^QDVDDVEESSAVRVRR^∧^GYGCPFNQYQCHSHCSGIRGYKGGYCKGLFKQTCTCY
OtdB	MKVLCFLLLLLLTGLLTSRA^*^AVLDTRRDPEDGT^‡^GNDCPHNEIACTLKCERDGFAYGRCTGLVLDQKCECIA
OtdC	MTPLRFSLVCFLVLSAVVTATA^*^FQLRSVHNTEH^‡^AYGCPGYRAMCRKHCVETFGFEGYCGGAHRNECKCRG
OtdD	MKIVLVLLVCVMAFGVHS^*^SPPAADGGSGY^‡^GNGCPSNPAQCNQHCLDTRDLTGHCKGYQMTFCDCGW

* Signal peptide cleavage site;

∧ propeptide cleavage site;

‡ *predicted mature peptide start site if there is no propeptide*.

**Figure 1:**
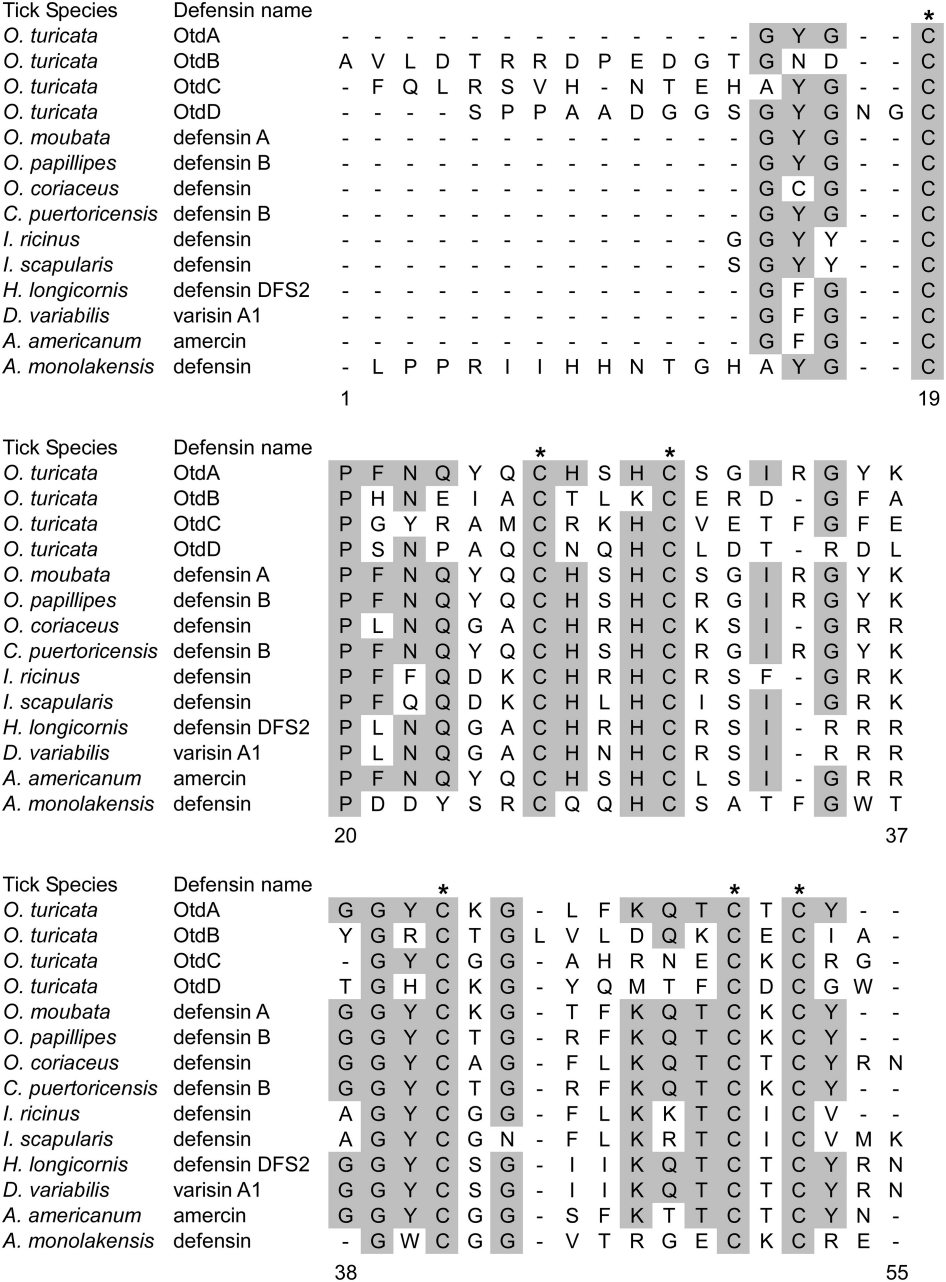
Alignment of mature tick defensin amino acid sequences. Amino acid sequences for the mature peptide of 10 tick defensins and our four putative defensins were obtained from NCBI and RACE sequencing, respectively. Alignment was performed using ClustalW in MEGAX 10.0.5. Shaded amino acids indicate amino acids that are shared among at least 50% of the sequences. Asterisks indicated the cysteines common to defensins. Numbers indicate the position of amino acids.

We used the ProP 1.0 server to predict potential cleavage sites within the defensin sequences and to identify the signal, pro-, and mature peptides (Table 2). OtdA was predicted to have both a signal peptide and propeptide, as indicated by the presence of a furin motif (RVRR), while OtdB, OtdC, and OtdD only had predicted signal peptides. No other *O. turicata* putative defensins contained the furin motif. The amino acid sequences of the mature putative defensins also were aligned to the sequences of 10 tick defensins from ixodids and argasids (Figure 1). Importantly, all four putative *O. turicata* defensins had six cysteines that aligned with the cysteines of other tick defensins (Figure 1). OtdA also had the most conserved amino acids (67.6%) of any of the *O. turicata* defensins when compared to the other proteins. OtdB had the fewest conserved amino acids (25.5%), while OtdC and OtdD had 27.1 and 31.3% amino acid identity, respectively. Except for *A. monolakensis*, all other tick defensin sequences had at least 46.2% of their amino acids identical, and 7 of 10 sequences had more than 50% amino acid identity.

**Figure 2:**
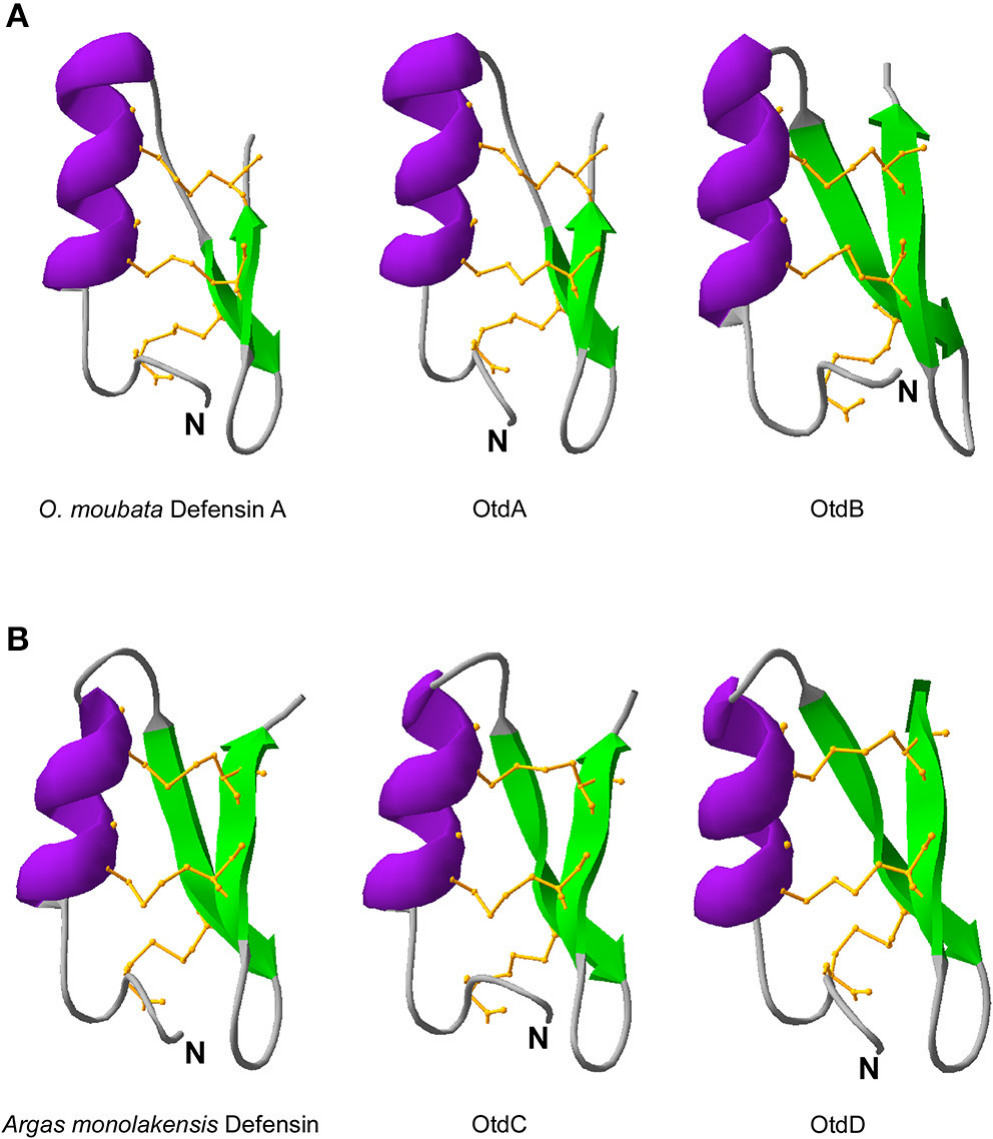
Predicted protein structure of defensins. In **(A)** are the protein structure of soft tick defensins *O. moubata* and the two most structurally similar *O. turicata* defensins, OtdA, OtdB. In **(B)** *A. monolakensis* defensin is shown with the most similar *O. turicata* defensins, OtdC and OtdD. Structures were predicted using MODELLER and visualized using SWISS-PDB viewer. Purple represents the alpha helix, green are the beta sheets, and the orange ball and stick regions are the disulfide bridges between cysteines C1–C4, C2–C5, and C3–C6. *N* represents the *N* terminus of the model.

Using protein modeling templates, tertiary structures of the putative *O. turicata* defensins were predicted. Included in the analyses were the tertiary structures for *O. moubata* defensin A and *A. monolakensis* defensin because they were the most similar to the *O. turicata* defensins (Figures 2A,B). All the evaluated defensins had an alpha helix followed by two beta sheets held together by three disulfide bridges. As is characteristic of arthropod defensins, it is predicted that the disulfide bridges form between C1–C4, C2–C5, and C3–C6. From the prediction, OtdA and *O. moubata* defensin A had longer alpha helices (11 amino acids) and shorter beta sheets (three amino acids) (Figure 2A). OtdB had a longer alpha helix (10 amino acids) and longer beta sheets (five amino acids) (Figure 2A). OtdC and OtdD had shorter alpha helices (nine amino acids) and longer beta sheets (five amino acids), which was similar to *A. monolakensis* defensin (alpha helix: eight amino acids, beta sheets: five amino acids) (Figure 2B).

### Phylogenetic Analysis of Arthropod Defensins

**Figure 3:**
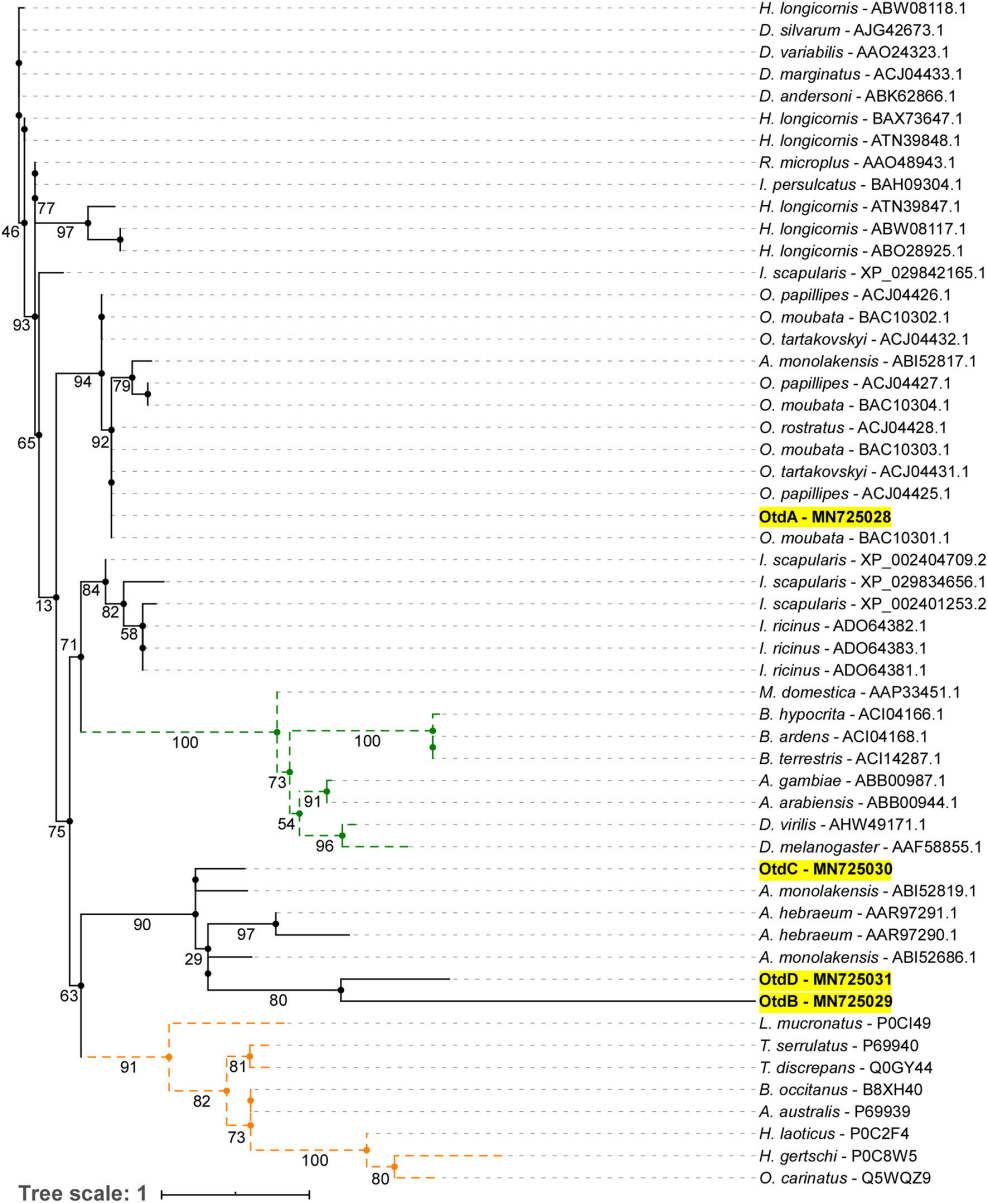
Phylogenetic tree of mature arthropod defensins. Maximum likelihood tree showing mature protein region of defensins from ticks and insects, and defensin-like scorpion toxins. Bootstrap values (1,000 bootstraps) are shown at the nodes. Names highlighted in yellow represent the *O. turicata* defensins (OtdA, OtdB, OtdC, and OtdD) introduced in this manuscript. Dotted orange lines represent scorpion toxin, defensin-like sequences, and dotted green lines represent insect defensin sequences.

A maximum likelihood (ML) analysis of tick, insect, and scorpion mature defensins and defensin-like toxins was generated to assess the evolutionary relationship between our novel putative *O. turicata* defensins and other arthropod defensins (Figure 3). The ML analysis showed a distinct clade for scorpion defensin-like toxins (orange), insect defensins (green), and four clades for tick defensins. OtdA was within a clade made up of *Ornithodoros* and *Argas* defensins. OtdB, OtdC, and OtdD clustered together in one clade with *Argas* and *Amblyomma* defensins.

### Expression of *O. turicata* Defensins in the Midgut and Salivary Glands of Post-molt and Fed Ticks

**Figure 4:**
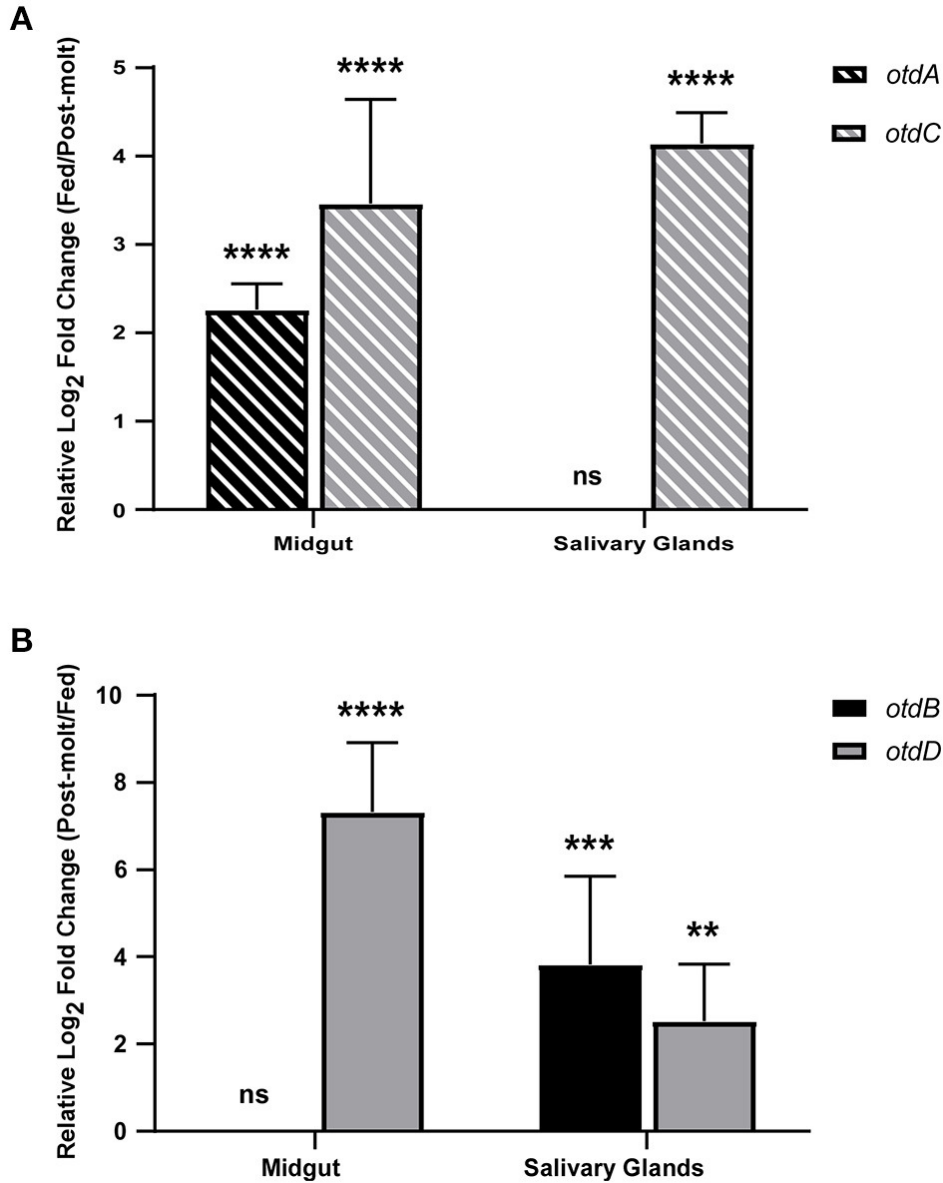
Expression analysis of putative *O. turicata* defensins in post-molt and fed midguts and salivary glands. Putative *O. turicata* defensins **(A,B)** expression was assessed in the fed and post-molt midguts and salivary glands. Defensin expression was normalized to β*-actin* expression and the relative log_2_ fold change was calculated using the 2^−ΔCt^ method with post-molt **(A)** or fed **(B)** tissues as the control. Statistical significance was calculated between post-molt and fed tissue using the Student's *t*-test with Welch's correction. (*P*-value: ** ≤ 0.01, *** ≤ 0.001, **** ≤ 0.0001).

Previous studies indicate midgut and salivary gland colonization is essential for pathogen transmission from *Ornithodoros* species (Hess et al., 1987; Lopez et al., 2011; Boyle et al., 2014; Krishnavajhala et al., 2017). Therefore, expression of the four putative defensins was further evaluated in pooled midguts and salivary glands 1 week after ticks fed and after they molted. Expression was considered significantly different if there was a log_2_ fold change of at least 1 and the *p* ≤ 0.05. In fed ticks (Figure 4A), *otdA* and *otdC* were up-regulated compared to post-molt *O. turicata. otdA* was expressed in the midgut of post-molt ticks and significantly up-regulated in the midgut within a week after feeding (log_2_ fold change = 2.26 ± 0.29) (Figure 4A). *otdA* was expressed in the salivary glands of fed and post-molt ticks and there was not a significant change in expression between the conditions. Also, we did not detect transcripts of *otdC* within 40 cycles in the midgut of post-molt ticks, but the gene was significantly up-regulated in fed ticks (log_2_ fold change = 3.46 ± 1.18) (Figure 4A). *otdC* was expressed in the salivary glands of post-molt ticks and was significantly up-regulated within 1 week after ticks fed (log_2_ fold change = 4.14 ± 0.35) (Figure 4A).

We also detected defensin genes to be up-regulated in post-molt ticks (Figure 4B). Transcripts of *otdD* were not detected in the midgut of fed ticks but the gene was up-regulated after ticks molted (log_2_ fold change = 7.32 ± 1.59) (Figure 4B). Moreover, this gene was expressed in the salivary glands of fed ticks and significantly up-regulated in post-molt *O. turicata* (log_2_ fold change = 2.52 ± 1.31) (Figure 4B). While there was no significant difference in *otdB* expression in the midgut between fed and post-molt ticks, the gene was significantly up-regulated in the salivary glands of post-molt ticks relative to fed ticks (log_2_ fold change = 3.82 ± 2.02) (Figure 4B).

## Discussion

The present study identified transcripts coding for annotated defensins from *O. turicata* and assessed their expression in the midgut and salivary glands of fed and post-molted ticks. Since the four putative defensin genes (*otdA, otdB, otdC*, and *otdD*) were initially identified in a salivary gland transcriptome from *O. turicata* (Bourret et al., 2019), we determined and validated their full sequences. Additionally, a computational analysis identified conserved motifs and the six cysteines characteristic of defensins. The expression patterns of these defensins in the midgut and salivary glands suggested they may have differing functional roles dependent upon whether the tick is fed or in the post-molted state. Collectively, these findings set the framework to further define soft tick immunity, an understudied aspect of vector biology.

Seminal work in *Ornithodoros* defensins was performed in the Old World species *O. moubata*. Van der Goes van Naters-Yasui and colleagues purified and determined the partial amino acid sequence of a small peptide (4 kDa) with homology to a scorpion defensin (Van Der Goes Van Naters-Yasui et al., 1999). Subsequently, Nakajima and co-workers identified four isoforms of this defensin that were over 78% homologous, constitutively produced in the midgut lumen of *O. moubata*, and up-regulated after ticks blood fed (Nakajima et al., 2001, 2002a,b). In *O. turicata*, OtdA was most homologous to *O. moubata* defensin isoform A. While additional isoforms of OtdA were not identified in our study, our transcriptional findings were consistent with the work performed in *O. moubata*. We detected *otdA* transcript in post-molt ticks, and upon feeding the gene was up-regulated in the midgut. Moreover, we expanded our investigation to assess *otdA* expression in the salivary glands, which is another tissue typically colonized by pathogens (Boyle et al., 2014; Krishnavajhala et al., 2017). Within the salivary glands *otdA* was expressed, but we failed to detect a change in transcript levels in response to blood feeding.

Our computational analyses indicated differences in amino acid motifs between the four identified defensins. Typically, arthropod defensin motifs include a signal peptide, a propeptide, and a mature peptide that consists of six cysteine residues that form three disulfide bonds (Bulet and Stöcklin, 2005). Furthermore, the propeptide is characterized by a furin motif that serves as a cleavage site. OtdA was most similar to other known tick defensins because it contained the signal peptide and propeptide. OtdB, OtdC, and OtdD lacked the propetide but retained the signal peptide. Furthermore, these proteins contain the necessary cysteines for disulfide bridge formation in the mature peptide, which is critical for microbicidal activity (Bulet and Stöcklin, 2005). Our findings suggest that not all defensins require the furin cleavage site for functionality, and that OtdB, OtdC, and OtdD likely form a mature peptide after the signal peptide is cleaved. In support of this, production of synthetic defensins only consists of the mature peptides, and they retain their functional activity (Nakajima et al., 2002b; Prinsloo et al., 2013; Malan et al., 2016).

While defensins are important in tick immunity, studies indicate that they possess a dual-function role in homeostasis. OsDef2, a defensin identified in *Ornithodoros savignyi*, was shown to have immune function and antioxidant properties acting as a scavenger for ROS and RNS, respectively) (Prinsloo et al., 2013; Malan et al., 2016). Previous work indicated that the midgut and salivary glands of *O. turicata* are nitrosative and oxidative environments, respectively (Bourret et al., 2019). Expression of two putative dual oxidase (*duox1* and *duox2*) genes in midgut tissue from *O. turicata*, and a single nitric oxide synthase (*nos*) gene, was expressed in salivary glands (Bourret et al., 2019). Immunofluorescent staining of *O. turicata* further validated transcriptional findings and determined the production of RNS in midguts and ROS in salivary glands (Bourret et al., 2019). Given these findings, determining a dual role of tick defensins in homeostasis and immunity is important for the development of control measures for these vectors and their respective pathogenic microbes.

Pathogens transmitted by *Ornithodoros* ticks have evolved to colonize this long-lived vector that completes blood feeding within minutes of attachment to the host. Vector competence studies in relapsing fever spirochete and ASFV models demonstrated persistent colonization of *Ornithodoros* midguts, and within ~10–20 days the pathogens colonize the salivary glands (Hess et al., 1987; Schwan and Hinnebusch, 1998; Boyle et al., 2014; Krishnavajhala et al., 2017). Once colonized, the tick remains infected for years (Davis, 1943; Hess et al., 1987). Consequently, the tick immune response likely plays a role in early pathogen colonization after blood feeding and during persistent infection in post-molt ticks.

**Figure 5:**
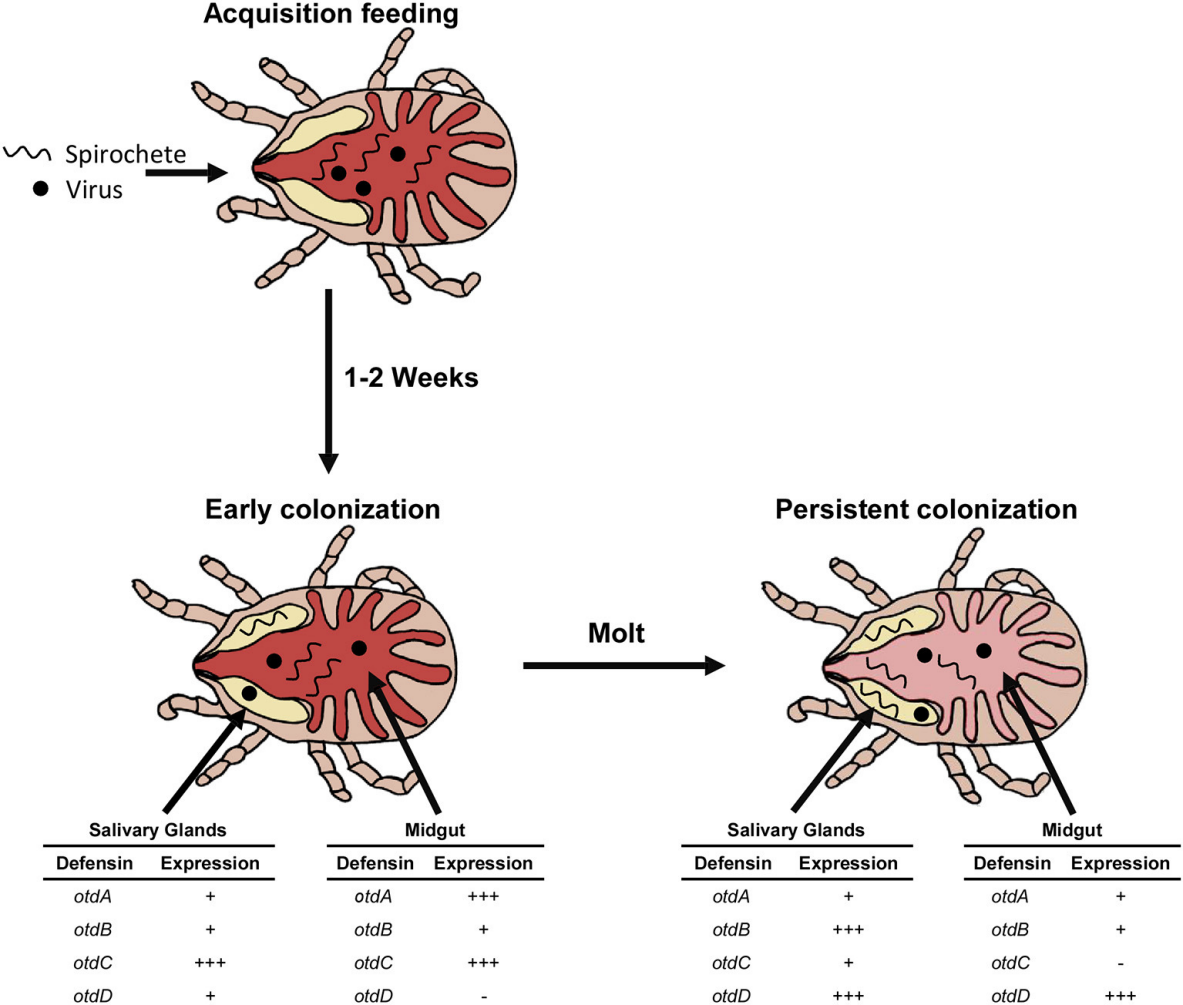
Model of pathogen colonization and defensin expression. Spirochetes and viruses are imbibed during the acquisition blood feeding and enter the midgut (red). Over the next 1–2 weeks a subset of the population migrates out of the midgut to colonize the salivary glands (yellow). At this time there is an upregulation (+ + +) of *otdA* and *otdC* in the midgut and *otdC* in the salivary glands. *otdB* is expressed (+) in the midgut. *otdD* is not expressed in the midgut (-) but transcript detected in the salivary glands with *otdA* and *otdB*. This characterizes early pathogen colonization of the tick. Persistent colonization occurs after the tick has molted and the pathogen remain in both the midgut (light red) and salivary glands. In the midgut *otdA* and *otdB* are expressed (+), *otdC* is not expressed (-), and *otdD* is upregulated (+ + +). In the salivary glands *otdA* and *otdC* are expressed (+), while *otdB* and *otdD* are upregulated (+ + +).

The expression kinetics of *otdA, otdB, otdC*, and *otdD* suggest functional roles of the proteins in early and persistent colonization, and our hypothesized model is shown in Figure 5. For example, *otdA* and *otdC* were significantly up-regulated in the midgut within a week after blood feeding, which suggests the proteins function in homeostasis and during early pathogen colonization. In the midgut of post-molt ticks, the up-regulation of *otdD* suggests the protein may function in maintaining pathogen load during persistent colonization.

Our study also evaluated defensin expression in salivary glands, which is an important tissue for pathogen maintenance and transmission. In these tissues, *otdC* was expressed in post-molt ticks and up-regulated within a week after blood feeding. We hypothesize that this gene may function in tick immunity during early microbe colonization of the salivary glands (Figure 5). Furthermore, persistent infection may be characterized by the up-regulation of *otdB* and *otdD*. While these genes were expressed 1 week after blood feeding, they were up-regulated after *O. turicata* molted.

With very little work focused on soft tick immunity and defensins, our study provides a basis to further define the molecular mechanisms of vector competence. While we assessed expression kinetics of four novel defensins in late stage *O. turicata* nymphs, additional studies should confirm expression at different tick life stages. Additionally, given that relapsing fever spirochetes and ASFV are transmitted transovarially, assessment of defensin expression in reproductive organs is an important aspect of vector biology and pathogenesis. Future work will also focus on the validation of our transcriptional findings at the protein level. We will also assess the bactericidal properties of the identified putative defensins on relapsing fever spirochetes and determine whether the pathogens have evolved mechanisms to modulate tick immunity. These studies will provide critical insight into the maintenance of pathogens in an understudied tick vector.

## Data Availability Statement

The datasets generated for this study can be found in the Genbank database with the following accession numbers: otdA (MN725028), otdB (MN725029), otdC (MN725030), and otdD (MN725031).

## Ethics Statement

Tick feedings were performed with mice in accordance with the Institutional Care and Use Committee (IACUC) at Baylor College of Medicine under protocol number AN-6563. The animal program at Baylor College of Medicine is compliant with standards and guidance established by the Association for the Assessment and Accreditation of Laboratory Animal Care and the Nation Institution of Health of Laboratory Animal Welfare. The animal husbandry team at Baylor College of Medicine provided all veterinary staff and animal care.

## Author Contributions

BA designed the study, performed experiments, analyzed data, and wrote the manuscript. ARK performed the experiments and data analysis. RM performed the phylogenetic analysis and wrote the phylogenetic analysis. AK performed experiments and assisted in data analysis. PT provided samples and provided experimental critique. AP contributed to phylogenetic analysis. JL designed the study, analyzed the data, and wrote the manuscript.

## Conflict of Interest

The authors declare that the research was conducted in the absence of any commercial or financial relationships that could be construed as a potential conflict of interest.
