# Corrigendum: Differential Expression of Putative *Ornithodoros turicata* Defensins Mediated by Tick Feeding

**DOI:** 10.3389/fcimb.2020.00310

**Published:** 2020-07-02

**Authors:** Brittany A. Armstrong, Alexander R. Kneubehl, Robert D. Mitchell, Aparna Krishnavajhala, Pete D. Teel, Adalberto A. Pérez de León, Job E. Lopez

**Affiliations:** ^1^Department of Molecular Virology and Microbiology, Baylor College of Medicine, Houston, TX, United States; ^2^Department of Pediatrics, National School of Tropical Medicine, Baylor College of Medicine, Houston, TX, United States; ^3^Knipling-Bushland U.S. Livestock Insects Research Laboratory, Veterinary Pest Genomics Center, Department of Agriculture—Agricultural Research Service, Kerrville, TX, United States; ^4^Department of Entomology, Texas A&M AgriLife Research, College Station, TX, United States

**Keywords:** *Ornithdoros turicata*, antimicrobial peptide (AMP), gene expression, defensins, argasid (soft) ticks, immune response

The original article stated that “Moreover, *O. turicata, O. coriaceus*, and *O. parkeri* were experimentally shown to be competent vectors of African swine fever virus (ASFV) (Hess et al., 1987), an emerging pathogen in Europe and Asia,” which is incorrect. It should read “Moreover, *O. turicata* and *O. coriaceus* were experimentally shown to be competent vectors of African swine fever virus (ASFV) (Hess et al., 1987), an emerging pathogen in Europe and Asia. *O. parkeri* was able to be infected with ASFV, but unable to transmit the pathogen via tick bite (Hess et al., 1987).” A correction has been made to the **Introduction**, first paragraph:

“*Ornithodoros* (argasid) species are vectors of veterinary and medically significant pathogens. The primary species in the United States that transmit pathogens include *Ornithodoros turicata, Ornithodoros hermsi, Ornithodoros parkeri, Ornithodoros talaje*, and *Ornithodoros coriaceus* (Davis, 1939; Cooley and Kohls, 1944; Hess et al., 1987; Donaldson et al., 2016; Lopez et al., 2016; Sage et al., 2017). These species have been implicated in the transmission of tick-borne relapsing fever spirochetes (Lane et al., 1985; Dworkin et al., 2002; Nieto et al., 2012; Lopez et al., 2016; Christensen et al., 2017; Bissett et al., 2018). Moreover, *O. turicata* and *O. coriaceus* were experimentally shown to be competent vectors of African swine fever virus (ASFV) (Hess et al., 1987), an emerging pathogen in Europe and Asia. *O. parkeri* was able to be infected with ASFV, but unable to transmit the pathogen via tick bite (Hess et al., 1987). *Ornithodoros* ticks play a significant role in pathogen maintenance, yet very little is known regarding vector competence.”

The authors apologize for the error and state that it does not change the scientific conclusions of the article in any way. The original article has been updated.
